# In Vivo Suppression of HIV by Antigen Specific T Cells Derived from Engineered Hematopoietic Stem Cells

**DOI:** 10.1371/journal.ppat.1002649

**Published:** 2012-04-12

**Authors:** Scott G. Kitchen, Bernard R. Levin, Gregory Bristol, Valerie Rezek, Sohn Kim, Christian Aguilera-Sandoval, Arumugam Balamurugan, Otto O. Yang, Jerome A. Zack

**Affiliations:** 1 Division of Hematology-Oncology, The David Geffen School of Medicine at UCLA, Los Angeles, California, United States of America; 2 Department of Microbiology, Immunology, and Molecular Genetics, The David Geffen School of Medicine at UCLA, Los Angeles, California, United States of America; 3 The UCLA AIDS Institute, The David Geffen School of Medicine at UCLA, Los Angeles, California, United States of America; 4 Division of Infectious Diseases, Department of Medicine, The David Geffen School of Medicine at UCLA, Los Angeles, California, United States of America; NIH/NIAID, United States of America

## Abstract

The HIV-specific cytotoxic T lymphocyte (CTL) response is a critical component in controlling viral replication *in vivo*, but ultimately fails in its ability to eradicate the virus. Our intent in these studies is to develop ways to enhance and restore the HIV-specific CTL response to allow long-term viral suppression or viral clearance. In our approach, we sought to genetically manipulate human hematopoietic stem cells (HSCs) such that they differentiate into mature CTL that will kill HIV infected cells. To perform this, we molecularly cloned an HIV-specific T cell receptor (TCR) from CD8+ T cells that specifically targets an epitope of the HIV-1 Gag protein. This TCR was then used to genetically transduce HSCs. These HSCs were then introduced into a humanized mouse containing human fetal liver, fetal thymus, and hematopoietic progenitor cells, and were allowed to differentiate into mature human CD8+ CTL. We found human, HIV-specific CTL in multiple tissues in the mouse. Thus, genetic modification of human HSCs with a cloned TCR allows proper differentiation of the cells to occur *in vivo*, and these cells migrate to multiple anatomic sites, mimicking what is seen in humans. To determine if the presence of the transgenic, HIV-specific TCR has an effect on suppressing HIV replication, we infected with HIV-1 mice expressing the transgenic HIV-specific TCR and, separately, mice expressing a non-specific control TCR. We observed significant suppression of HIV replication in multiple organs in the mice expressing the HIV-specific TCR as compared to control, indicating that the presence of genetically modified HIV-specific CTL can form a functional antiviral response *in vivo*. These results strongly suggest that stem cell based gene therapy may be a feasible approach in the treatment of chronic viral infections and provide a foundation towards the development of this type of strategy.

## Author Summary

There is a desperate need for the development of new therapeutic strategies to eradicate HIV infection. HIV actively subverts the potent natural immune responses against it, particularly cellular cytotoxic T lymphocyte (CTL) responses. The development of a therapy that allows long-lived immune self-containment of HIV and restoration of these CTL responses by the host would be ideal. Through genetic manipulation of human blood-forming stem cells, we introduced a molecule– an HIV-targeting T cell receptor (TCR)–that allowed the generation of functional HIV-specific CTLs following differentiation within human tissues in a humanized mouse model. To assess if these newly developed, HIV-specific CTLs can allow active suppression of HIV replication, we infected these mice with HIV. We found that the development of genetically modified, HIV-specific CTLs in these mice results in the presence of a functional antiviral CTL response *in vivo* that significantly lowers viral replication following HIV infection. These results have strong implications for the use of this technology to engineer the human immune response to combat viral infections and suggest that genetic engineering via HSCs may allow tailoring of the immune response to target and eradicate HIV.

## Introduction

Human hematopoietic stem cells (HSCs), through development in the thymus, are capable of producing progeny T cells that generally display one of a vast repertoire of T cell receptors (TCRs). In the case of many non-persistent viral infections, T cells bearing TCRs specific to viral antigens mediate a potent antiviral response that results in the clearance of the virus from the body. Even in the presence of most persistent viral infections, a potent T cell response is mounted; however it often fails to clear the virus from the body. A critical component of the T cell antiviral response is the CD8+ cytotoxic T lymphocyte (CTL), whose primary function is to recognize viral antigens (in the context of human leukocyte antigen class I (HLA I)) and kill virus-infected cells. In HIV infection, the potent antiviral CTL response is critical for establishing relative control of viral replication during the acute and chronic infection stages of the disease [1]–[6]. However, unlike what is observed in most non-persistent viral infections, the CTL response fails to clear HIV from the body. The magnitude, breadth, functional quality, and kinetics of the antiviral CTL response all are critical in controlling ongoing viral replication; however, the reasons for the failure to rid the body of virus are not completely understood [7], [8]. Ongoing viral replication and viral evolution in the infected host is one important, although highly confounding, factor in the persistence of HIV in chronic infection [4], [5]. Even under effective antiretroviral therapy (ART), the virus is not cleared from the body and the level of HIV specific CTLs declines, likely due to lower levels of antigen to stimulate the persistence/generation of these cells [9], [10]. Due to the importance of T cell responses in controlling and eliminating viral infection there exists a great need to explore ways to enhance antiviral T cell immune responses.

Recently, much of attention in HIV research has focused on ways to enhance or correct the defects in HIV-specific CTL responses. Gene therapy-based approaches that augment immunity towards viral antigens represent unique, yet largely unexplored, strategies towards the treatment of HIV disease. We have previously examined the feasibility of a stem cell-based gene therapy approach to enhance cell-mediated immunity towards chronic HIV infection. In these studies, we demonstrated that human HSCs genetically modified with genes encoding a human HIV-specific TCR can produce mature, fully functional T cells in human thymus implants in severe combined immunodeficient (SCID) mice. The resulting genetically directed CD8+ T cells are capable of killing HIV antigen-expressing cells *ex vivo* [11]. Further, we showed that the appropriate restricting human leukocyte antigen (HLA) class I molecule is required for proper development of transgenic TCR-containing CTLs. In all, our earlier studies demonstrated that TCR-modified human HSCs can be directed to develop into mature CTLs in a human thymus environment in the context of the proper HLA type. However, as the SCID-hu mouse model demonstrated poor peripheral reconstitution and function of human immune cells, these studies did not address the ability of these cells to suppress HIV replication *in vivo*.

**Figure 1:**
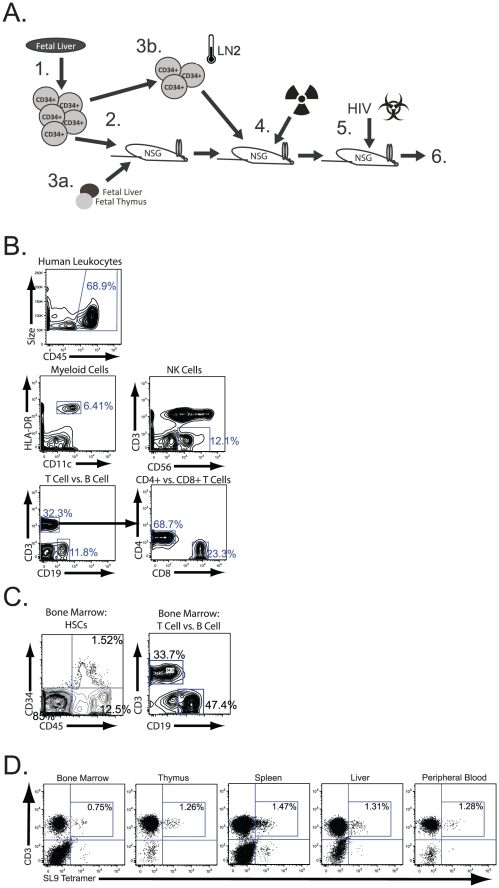
Construction and multilineage reconstitution of NSG-CTL mice. **A.** Schematic illustrating the construction of NSG-CTL mice: CD34+ cells are isolated from fetal liver by cell sorting (1). then are transduced with lentiviral vector containing the SL9-specific TCR (2). A fraction of these cells are then implanted under the kidney capsule in NSG mice flowing combination with fetal liver stromal elements and fetal thymus in matrigel (3a). Another fraction of these transduced cells are viably frozen in liquid nitrogen (LN2) (3b). Three weeks following implantation, the engrafted mice are then sublethally irradiated (3 Gy) and previously frozen cells are thawed and injected intravenously into these mice, where the cells home to and engraft in the bone marrow (4). 6–12 weeks following injection of cells, TCR expression was analyzed and mice were infected with HIV (5). Mouse blood is then assessed for HIV infection 2 and 6 weeks following infection (6). **B.** Multilineage hematopoietic reconstitution of NSG mice receiving genetically modified HSCs. Peripheral blood from these mice were assessed by flow cytometry and gated for CD45+ human leukocytes (top panel). These cells were assessed for the denoted cell surface marker expression including HLA-DR+CD11c+myeloid cells, CD3-CD56+ NK cells, CD3+ T cell, CD19+ B cells. CD3+ cells were gated (lower left panel) and assessed for CD4 and CD8 expression (lower right panel). The numbers indicate the percentage of each population of cells in the mouse peripheral blood. **C.** Repopulation of HSCs in mouse bone marrow. Mouse bone marrow was assessed for the presence of CD34+ human HSCs (left panel) and CD3+ T cell and CD19+ B cell engraftment 6 weeks following CD34+ cell injection by flow cytometry. **D.** Reconstitution of NSG-CTL mice with cells expressing the HIV-specific TCR transgene. Cells were isolated from the indicated organ in NSG-CTL mice 6 weeks following CD34+ cell injection and analyzed by flow cytometry for CD3+ T cells binding SL9-containing tetramers. The numbers indicate the percentage of T cells within the indicated organ expressing the transgenic TCR. The data are representative of mice receiving human tissue and HIV-specific TCR transduced CD34+ cells in the same experiment identified above (n = 12).

In the present studies, we examined the ability of genetically modified T cells derived from HSC transduced with a single HIV-specific TCR to suppress viral replication *in vivo*. We utilized a modified version of a newly established humanized mouse model, the non-obese diabetic (NOD)-SCID, common gamma chain knockout (γc−/−), humanized bone marrow, fetal liver and thymus (the NSG-BLT) mouse model, which allows the generation of peripheral human immune responses, and serves as an effective model for HIV infection and pathogenesis [12]–[14] (see Figure 1A). These humanized mice display multilineage human hematopoiesis and systemic engraftment of peripheral organs with human blood cell types including T lineage cells, B lineage cells, myeloid lineage cells, NK cells, as well as cells from other lineages [12] (and see Figure 1B). We modified human hematopoietic stem cells in this model with molecularly cloned genes corresponding to a TCR specific to the HIV-1 Gag 77–85 SLYNTVATL (SL9) epitope to allow the production of mature HIV-specific CTLs in multiple organs of these reconstituted mice. We determined that human T cells that expressed the HIV-specific TCR were capable of suppressing HIV replication *in vivo* and preventing or slowing viral damage to the engrafted human immune system. These studies establish a system to examine “genetic vaccination” approaches that target chronic viral infection and to more closely examine mechanisms of human antiviral immunity *in vivo*.

## Results

### Genetic modification and multilineage human hematopoiesis *in vivo*

We have previously demonstrated that human hematopoietic stem cells can be genetically modified by delivering a gene for an HIV-specific TCR, and develop into mature T cells in an HLA-restricted fashion in the human thymus of SCID-hu mice [11]. These newly produced, SL9 gag antigen-specific, naive T cells were determined to be capable of producing IFN-γ in response to peptide stimulation and were found to be lytic to SL9 peptide loaded target cells. However, it was not known whether these genetically modified HIV-specific CTLs could traffic to relevant organs in the mice and whether they were capable of killing HIV infected cells *in vivo*. To address this question, we established an improved model, based on the NSG-BLT model previously shown to allow HIV replication [15], [16], as a surrogate system to assess the antiviral efficacy of engineered, HIV-specific T cells *in vivo*. NSG mice were implanted with human fetal liver-derived **C**D34+ HSCs that had been modified with a lentiviral vector containing the genes for a TCR targeting the HIV Gag SL9 epitope, or as a control, with HSCs modified with a lentiviral vector containing a non-HIV-specific TCR with unknown specificity. In addition, these mice received implantation of human fetal **T**hymus and **L**iver under the kidney capsule to facilitate human T cell development. Hence, we term this the NSG-CTL model (Figure 1A).

As genetic manipulation of HSCs is required in this model, we initially determined the effects on this type of lentiviral transduction on multilineage hematopoietic potential of HSCs in the humanized mice. Phenotypic markers of human hematopoiesis were examined by flow cytometry in mice within 6 weeks following implantation of human tissues. One hundred percent of the mice receiving human tissue had human cells in the peripheral blood, including myeloid, natural killer (NK), T cell, and B cell lineages (Figure 1B). In these mice, the average percentage of human CD45+ cells in the peripheral blood was 53% of the total cells (with a standard deviation of 29% and a range of 19%–80%, n = 12). We more closely examined the bone marrow in these mice for the presence of human cell engraftment, particularly human HSC engraftment. We found a significant population of human CD34+ HSCs in the bone marrow (Figure 1C). The majority of these cells coexpressed the CD45 molecule, which is indicative of cells with lymphopoietic potential [17]. In addition, there were significant populations of both CD3 expressing T cells and CD19 expressing B cells in the bone marrow of these mice. This indicated that multilineage human hematopoiesis occurs in these mice and provides evidence that, in addition to T cells, other components of the human immune system are present. These data demonstrated that our modification of the NSG-BLT humanized mouse utilizing genetically modified human hematopoietic stem cells does not negatively affect human hematopoiesis.

We then examined the animals for the presence of cells expressing the transgenic, HIV specific TCR by MHC tetramer staining. We found CD3+ T cells expressing the transgenic TCR in all organs assessed, including the bone marrow, thymus, spleen, liver, and peripheral blood of the mice receiving transduced human hematopoietic stem cells (Figure 1D). Thus, we have observed long-term, multilineage human immune reconstitution and the development of mature T cells that express the transgenic, HIV-specific TCR in multiple organs in the NSG-CTL mouse.

### Suppression of HIV replication and CD4 cell depletion *in vivo*

**Figure 2:**
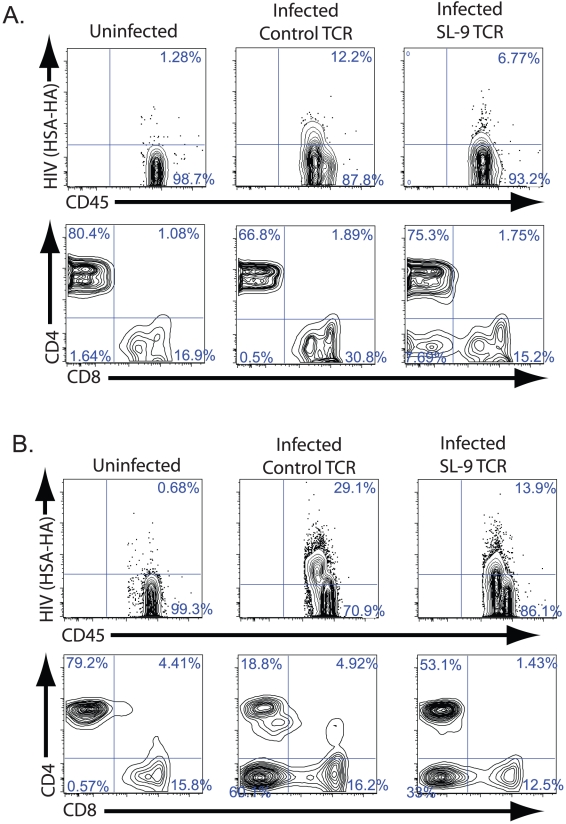
Suppression of viral replication in cells in the peripheral blood. Mice receiving the HIV Gag SL-9 specific TCR (left and right panels) or the non-specific control TCR (middle panels) were either left uninfected (left panels) or were infected with HIV-1_NL-HSA-HA_ (right and middle panels) and assessed for HIV (HSA-HA) expressing, human CD45+ cells (top panels) and CD4 and CD8 expression by gated CD3+ T cells (bottom panels). Mice were assessed by flow cytometry for these cell surface markers **A.** 2 weeks, and **B.** 6 weeks post infection. The numbers indicate the percentages of cells that exist with each of the respective quadrants. The data are representative of the phenotypic profile of 1 mouse of 6 mice receiving the SL-9 specific TCR, and 1 mouse of 6 receiving the control TCR and are representative of 3 separate experiments with a minimum of 3 mice in each experimental group.

**Figure 3:**
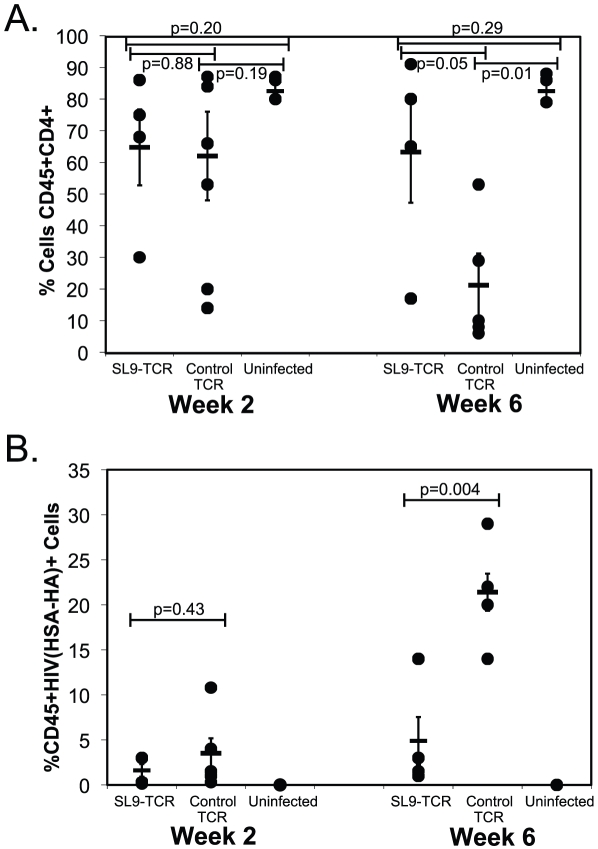
Suppression of HIV replication by HIV-TCR containing T cells. **A.** Suppression of HIV-1 induced CD4 cell depletion by cells containing HIV-specific TCR. Mice containing cells derived from HSC transduced with either HIV Gag SL9-epitope specific TCR (n = 6 mice) or a non-specific control TCR (n = 7 mice) were infected with HIV-1_HSA-HA_ or left uninfected (n = 4 mice)(SL9-specific TCR containing mice) and assessed 2 weeks and 6 weeks following infection for peripheral human CD45+, CD4+ cells. Statistical comparison of CD4 cell depletion to uninfected controls was performed using the Student's t test, p values are provided for each indicated comparison. The solid lines represent the mean +/− the standard error of the mean (SEM). **B.** Suppression of HIV expressing cells. Mice treated as described in (A) were assessed for human CD45+ cells expressing HIV by flow cytometry for the HSA-HA marker gene. Comparison of HIV expression levels between SL-9 containing and control TCR-containing mice are provided at week 2 and week 6 post infection (Student's t test). The data represent 1 experiment of 3, with a minimum of 3 mice per experimental condition, and is a separate experiment than that depicted in Figure 2.

To assess if peripheral cells resultant from human hematopoietic stem cells that expressed the recombinant SL9-specific TCR were capable of suppressing HIV replication *in vivo*, NSG-CTL mice containing the HIV specific TCR or a control TCR were infected with HIV-1_NL-HSA-HA_. HIV-1_NL-HSA-HA_ is an engineered variant of HIV-1_NL4-3_ that contains the murine heat stable antigen (HSA) reporter gene modified to contain an Influenza hemagglutinin (HA) antibody epitope, which is cloned into the open reading frame of the vpr gene to allow detection of HIV-infected cells by cell surface detection of HA expression using flow cytometry [18]. Peripheral blood was assessed for the level of productively infected cells two and six weeks post infection. Within 2 weeks post infection, we observed a reduced level of productively infected cells in mice containing the HIV-specific TCR versus mice containing the control TCR (Figure 2A). In addition, there was less initial CD4 depletion in mice containing the HIV-specific TCR versus mice containing the control TCR. Within six weeks post infection, while there was an overall increase in virus-expressing cells from the earlier time point, we observed a marked reduction in productively infected cells in mice containing the HIV specific TCR versus the control TCR, indicating suppression of viral replication over time (Figure 2B). At this time point, mice containing cells expressing the HIV-specific TCR had a greater preservation of CD4+ T cells and higher CD4 to CD8 T cell ratios when compared to mice expressing the control TCR. Amongst all mice in the experiment, there was no statistically significant difference 2 weeks following infection with either CD4 cell count or with the percentage of cells expressing HIV, however there was a trend towards better preservation in CD4+ cell numbers as well as lower levels of virus-expressing cells in mice containing the HIV-specific TCR (Figure 3). However, by 6 weeks post-infection, there was a statistically significant difference in CD4 cell numbers and levels of infected cells between mice with cells expressing the HIV-specific TCR and mice with cells expressing the control TCR. Thus, genetic modification of HSCs with a single HIV-specific TCR produces peripheral T cells capable of suppressing cellular HIV expression and CD4 depletion *in vivo*.

### HIV-specific TCR suppression of plasma viremia *in vivo*

**Figure 4:**
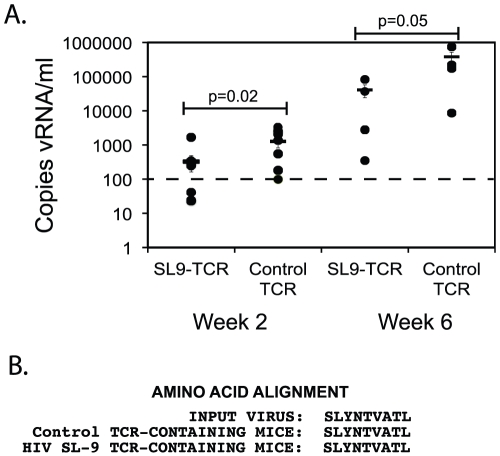
Suppression of HIV and viral evolution in the plasma of NSG-CTL mice. **A.** Blood plasma from the same HIV-1_HSA-HA_ infected mice as described in Figure 3 was collected 2 weeks and 6 weeks post infection. Viral RNA (vRNA) levels per sample (typically 50 µl of plasma per mouse) were determined by quatitative reverse transcriptase (RT)-PCR and results were multiplied by a standard factor to yield copies of vRNA per milliliter (ml) of blood. The points represent the copies of HIV vRNA per milliliter (ml) of blood and the solid line represents mean per group (+/− SEM). Statistical comparison was performed between SL9-specific TCR containing mice and non-specific TCR-containing mice and p values are provided (Student's t test). The dotted line indicates the limit of detection of the assay. The data are representative of 3 separate experiments, with a minimum of 3 mice per experimental condition, and utilized the same mice depicted in the experiment described in Figure 3. **B.** Evolution of the SL9 epitope in infected mice. Viral stock of the input virus, and virus from plasma of mice containing the control TCR or virus from plasma of mice containing the SL9-specific TCR 6 weeks following infection was sequenced utilizing a RT-PCR technique. The translated sequence is provided for each sample, with the SL9 epitope highlighted in bold.

We next sought to determine if cells modified with an HIV-specific TCR could suppress virus levels in peripheral blood plasma. However, quantitating plasma viremia in the mouse model is difficult due to the amount of plasma obtained per blood draw (typically ∼50 microliters), the limit of detection obtainable with this amount of blood, and the high cost associated with commercial assays. Therefore to measure viremia in this system, we developed a novel quantitative PCR-based technique for HIV in mouse plasma. Based on the recently elucidated secondary structure of the HIV genome [19], primers were designed to specifically target relatively “open” regions of the RNA genome that contain minimal secondary structure to attempt to allow increased sensitivity to detect viral RNA. Utilizing this technique, which has a reliable sensitivity of 5 copies of HIV RNA per sample, we determined that the viral load 2 weeks and 6 weeks post infection was significantly lowered in mice receiving the HIV-specific TCR versus mice receiving cells transduced with the control TCR (Figure 4A). This suggested systemic suppression of HIV replication *in vivo*. Surprisingly, analysis of the viral RNA for mutations in the SL9 epitope did not reveal the presence of any mutations in this epitope in the majority quasispecies, which was identical in comparison to the sequence of the input virus and the virus of infected mice containing the non-specific TCR control (Figure 4B). This suggested that in this period of time, viral escape to the selective pressure of the SL9 specific TCR had not occurred in the blood of these mice, possibly due to limited viral replication in this model. Thus, there was significant suppression of viral replication *in vivo* in mice expressing the HIV-specific TCR versus the control TCR and this suppression did not result in significant viral escape within 6 weeks following infection.

### TCR-engineered suppression of HIV in multiple lymphoid organs

**Figure 5:**
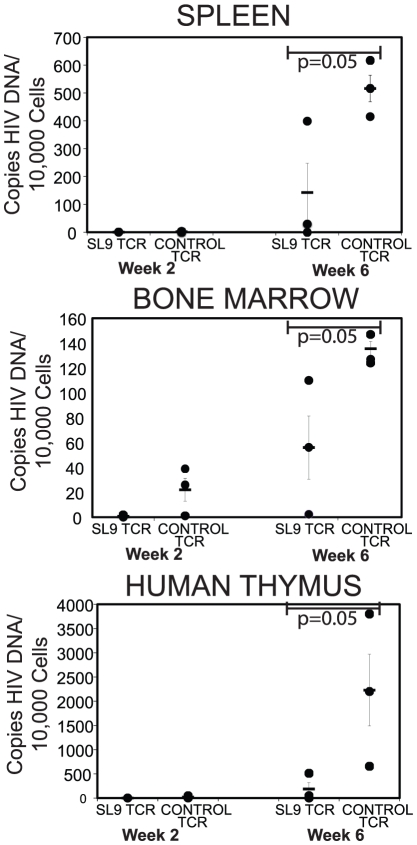
Suppression of HIV in multiple lymphoid compartments in NSG-CTL mice. HIV proviral DNA was quantitatively assessed in human cells from mouse spleen (top panel), bone marrow (middle panel), and human thymus implant (bottom) (n = 3 mice per group) 2 weeks (left side) and 6 weeks (right side) post infection. The points represent the copies of HIV proviral DNA per 10,000 human cells and the solid line represents mean per group (+/− SEM). Statistical comparison and p values were calculated using the WRST. The data represent 1 experiment of 2 separate experiments where these multiple organs were analyzed with a minimum of 3 mice per experimental condition and is derived from the same experiment depicted in Figures 3 and 4.

As illustrated in Figure 1, T cells expressing transgenic HIV-specific TCRs were found in multiple organs in mice receiving genetically modified HSCs. Based on this, we next addressed suppression of HIV in multiple organs in the lymphoid compartment in mice containing cells expressing the HIV-specific TCR. NSG-CTL mice that had received HSCs transduced with the HIV SL9-specific TCR or, separately, the non-specific control TCR were infected with HIV-1_NL-HSA-HA_. Sets of infected animals were then assessed 2 weeks and 6 weeks post infection for the quantity of HIV proviral DNA sequences in human cells in the spleen, bone marrow, and human thymus implant (Figure 5). We observed significant suppression of HIV replication in human cells in these organs as early as 2 weeks post infection (in the bone marrow) in mice receiving HSC containing the HIV-specific TCR. 6 weeks post-infection, HIV levels were significantly lower in the spleen, bone marrow, and human thymus implant in animals receiving the HIV-specific TCR as compared to mice receiving the control TCR. In addition, analysis for proviral DNA in human cells in the pooled peripheral blood cells (n = 3 mice per treatment group), revealed a similar trend, with 37 copies and 529 copies of HIV per 10,000 human cells at 2 weeks and 6 weeks post infection respectively, in mice containing the HIV-specific TCR, and 356 copies and 792 copies of HIV per 10,000 human cells at 2 weeks and 6 weeks, respectively, in mice containing the control TCR. Thus, these data indicate that there is significant suppression of HIV in multiple lymphoid tissues in animals receiving HSCs genetically modified to produce cells that specifically target HIV infected cells.

### Effector function of TCR-engineered CD8+ T cells and viral control *in vivo*

**Figure 6:**
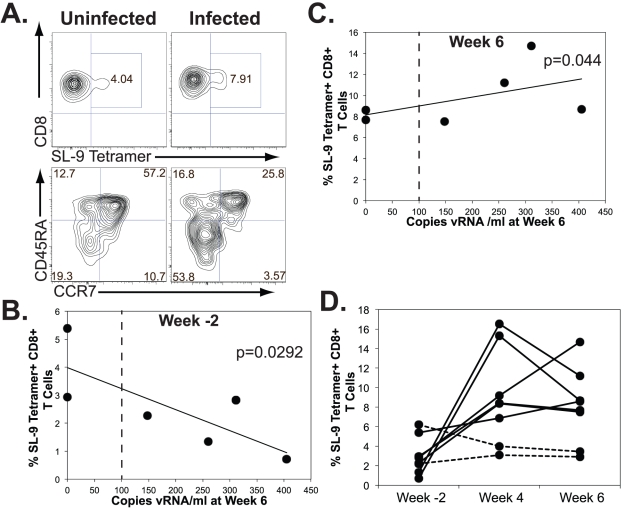
Effector cell differentiation and control of viral replication. **A.** Peripheral blood from uninfected (left column) and HIV infected (right column) mice was analyzed six weeks post infection with HIV for expression of CD8, the transgenic HIV-specific TCR (SL-9 Tetramer), and the CD45RA and CCR7 differentiation markers. The top row displays gated CD8+ T cells expressing the transgenic HIV-specific TCR. CD8+ cells expressing the transgenic TCR are indicated in the box gate and the percentage of total CD8+ cells expressing the transgenic TCR is provided in the gate. The bottom row displays the CD45RA versus CCR7 staining profile of the CD8+, SL-9 tetramer+cells in the gates indicated in the top row with the percentage of cells in each quadrant provided in their respective quadrants. **B.** Levels of transgenic HIV-specific TCR+, CD8+ T cells reconstituting mice 2 weeks prior to HIV infection versus viral load 6 weeks following HIV infection. The levels of SL-9 tetramer+cells of the CD8+ T cell population in individual mice were assessed in peripheral blood 2 weeks (week -2) prior to HIV infection by flow cytometry and are provided on the y-axis. The x-axis indicates the serum viral loads of these individual mice 6 weeks following infection. The data significantly correlate as determined by the SRCT and the p value is provided. Note that initially high levels of immune reconstitution correlate with lower viral loads at the 6 week time point. The dotted line indicates the limit of detection of the assay. **C.** Levels of transgenic HIV-specific TCR+, CD8+ T cells versus viral load 6 weeks following HIV infection. Data were analyzed as described above 6 weeks post HIV infection. The data significantly correlate as determined by the SRCT and the p value is provided. Note that at this time point, higher levels of CTL in the blood are found in animals with higher viral load. The dotted line indicates the limit of detection of the assay. **D.** Antigen-driven expansion of CD8+, HIV-specific TCR expressing cells in HIV infected mice. Levels of SL9 tetramer staining, CD8+ T cells were assessed in the peripheral blood of infected animals (solid lines) or uninfected animals (dashed lines) two weeks prior to infection (week -2), and 4 and 6 weeks post infection. Data is expressed as the percentages of tetramer+cells of total CD8+ T cells. Note that the level of HIV-specific cells in animals showing initially low levels of reconstitution are considerably higher at the late time point, suggesting proliferative response to the high levels of antigen. The data are representative of 1 of 3 separate experiments with a minimum of 3 mice per experimental condition.

We assessed the antiviral effector function of CTLs expressing HIV-specific transgenic TCRs in mice receiving genetically modified HSCs. In an additional series of experiments, mice containing the SL-9 specific TCR were infected with HIV or left uninfected and cells from the peripheral blood were assessed for phenotypic changes that would suggest differentiation. HIV infection resulted in phenotypic differentiation of HIV specific cells, as determined by SL9 MHC tetramer staining, into cells possessing an effector phenotype [20], [21](CD8+SL9Tetramer+CD45RA-CCR7-)(Figure 6A). This was similar to the phenotypic changes we observed in previous studies following *ex vivo* peptide stimulation of SL9-specific, TCR transgenic thymocytes [11] and in vivo responses to the MART-1 tumor antigen by MART-specific CD8 cells [22]. This increased loss of CD45RA and CCR7 expression that we observed in HIV- specific TCR-expressing cells in infected mice versus uninfected mice is indicative of antigen-specific induction of cellular differentiation. We then more closely analyzed the differences we observed viral suppression by and expansion of HIV-specific CTLs *in vivo* in infected mice. We found a significant correlation between the highest levels of reconstitution of HIV-specific TCR-expressing cells prior to infection and more effective suppression of viral loads in the serum six weeks following infection (Figure 6B). Interestingly, we noted that at six weeks following infection, mice that had greater levels of HIV-specific TCR-expressing cells in the peripheral blood had higher viral loads at this time point (Figure 6C). In addition, we saw significant antigen-driven expansion of HIV-specific TCR-expressing CTLs in infected animals compared to controls, with the greatest levels of expansion seen in animals with the lowest initial (week -2) transgenic TCR reconstitution (Figure 6D). Taken together, these results suggest that greater initial reconstitution of transgenic HIV-specific cells is more effective at controlling early viral replication. Furthermore these data suggest that the higher resultant viral loads in animals with initially low human immune reconstitution drive greater antigen-specific cell expansion over time. Thus, CTLs expressing the HIV-specific TCR undergo antigen-driven phenotypic differentiation and expansion in this model, which correlates with control of viral replication.

## Discussion

The CTL response has a pivotal role in controlling HIV replication in infected individuals. While HIV generates a potent natural immune response during the acute stage of infection, this response does not result in the control of viral replication or clearance of the virus from the body [4]–[6]. There are critical defects in the CTL response that result during chronic viral infection. These defects include the inadequate generation of a functional response due to low antigen-specific precursor frequency, expression of functional inhibitory molecules such as programmed death-1 (PD-1) and T-cell immunoglobulin domain and mucin domain 3 (TIM-3), and Cytotoxic T-Lymphocyte Antigen 4 (CTLA-4), and activation of suppressor cell activity [23]–[26]. In addition, HIV can directly or indirectly perturb viral antigen presentation, immunoregulatory cytokine production, T cell differentiation and effector/memory generation, and can infect CTLs themselves [27]–[33]. The maintenance of a potent antiviral CTL response is critical in all stages of infection and there are strong associations between the preservation of CTL responses specific for more conserved HIV epitopes, greater control of viral replication, and slower disease progression [5], [6].

In the present study, we demonstrate the feasibility of engineering human hematopoietic stem cells to become peripheral T cells capable of targeting HIV replication *in vivo*. Our previous studies provided evidence that the genetic modification of human hematopoietic stem cells with a lentiviral vector containing an antigen-specific TCR (specific to the SL9 Gag epitope) allowed the development of functional human T cells in human thymus implants in SCID-hu mice [11]. While this study demonstrated that transgenic TCR-containing T cells are capable of developing in the human thymus, the ability of these cells to target and kill HIV infected cells *in vivo* was not known. In the present study, we use an improved chimeric mouse model exhibiting a high degree of human immune cell reconstitution to significantly extend these observations to demonstrate that mature T cells expressing an antigen-specific human TCR are capable of developing and migrating to peripheral organs *in vivo*. In contrast to the SCID-hu Thy/Liv model, which is an excellent model for studies examining human thymopoiesis but limited in examining peripheral immune responses [34], we utilized a variation of the humanized BLT mouse model utilizing the NSG strain that allows multilineage hematopoiesis and human cell repopulation in peripheral organs [35], [36]. The generation of natural immune responses to HIV in these systems appears to be relatively limited, particularly the ability of these mice to elicit HIV specific human T cell responses which is likely due to incomplete human immune cell reconstitution, particularly antigen presenting cell reconstitution, to the levels seen in humans [12], [36], [37]. In addition, lower antigen-specific cell precursor frequency and the lack of or lower levels of human-specific cellular support immune components (such as costimulatory or immunoregulatory molecules, adhesion molecules, and cytokines) likely contribute to the lower levels of antiviral immune responses generated in humanized mice. The incomplete and varied immune reconstitution in the current humanized mouse systems results in differences in immune responses and kinetics of viral pathogenesis compared to natural HIV infection in humans. The reasons for this are unclear and vary between the different types of humanized mouse models; however, there are many similarities and parallels between HIV infection in humanized mice and humans which makes these surrogate models very powerful in their ability to allow the close examination of many aspects of HIV infection, transmission, pathogenesis, immunity, and therapeutic intervention [36]. While natural antiviral T cell immune responses are limited in current humanized mouse models, our studies suggest that the genetic “programming” of HSCs to produce T cells specific for HIV can overcome this limitation in this system and produce measurable T cell responses that have a significant antiviral effect *in vivo*. Further, we found it startling that the use of a single HIV-specific TCR can result in significant HIV suppression while natural suppressive antiviral CTL responses are polyclonal. These observations can provide the platform for future studies that allow the closer examination of the generation of human antiviral immune responses and the identification of factors involved in the persistence and potential eradication of HIV infection.

Previous attempts utilizing a gene therapy approach towards enhancing antigen specific cellular immune responses have focused on “redirecting” mature T cells towards viral or cellular antigens [38]–[46]. In these cases, genes for HIV-specific T cell receptors (TCRs) or chimeric antigen specific receptors were utilized to modify mature T cells to specifically target virus infected cells or malignancies. In some cases of the latter, tumor regression has occurred in treated individuals [45]–[47], which suggests that the genetic modification of T cells towards a specific antigen is feasible *in vivo* in humans and alludes to the potential for the further development of these strategies to target other diseases. However, the modification of mature T cells has several limitations, including the possibility of endogenous TCR miss-pairing with the newly introduced TCR, the development of intrinsic functional defects and/or the alteration of cellular effector/memory maturation pathways in the cells following heavy ex-vivo manipulation [47], and the maintenance of long-lived fully functional cells. A stem cell-based approach where HSCs are modified with an antigen specific receptor, however, may abrogate these complications by allowing the long term, continual natural development of mature T cells that bear the transgenic antigen-specific molecule. Normal development of these cells in the bone marrow and selection in the thymus would reduce the possibility of producing cells that are autoreactive through TCR miss-pairing and functionally altered through *ex vivo* manipulation, major drawbacks of mature T cell modification. We have recently shown that genetic modification of human HSC with a TCR specific for human melanoma allows the generation of melanoma-specific human T cells capable of clearing tumors in BLT mice [48]. Our current studies extend this type of approach to demonstrate the *in vivo* efficacy of TCR-modified stem cells to generate antigen-specific T cells that target a rapidly replicating viral infection *in vivo*. Our results document the ability of the resulting HIV-specific CTLs to dramatically reduce viral replication and consequent CD4 cell loss in a relevant model of HIV pathogenesis.

Recent stem cell-based attempts at protecting cells from direct infection by HIV through the modification of HSCs with antiviral genes or genes that knock down viral coreceptors [16], [49], [50] require high percentages of HSCs to be genetically modified to be protected from infection. Our results suggest that the approach of genetically vaccinating cells to target HIV infection would require much lower levels of genetic modification. Modification of human HSCs with a transduced TCR results in significantly increased naïve, antigen specific precursors. This level of transduction is sufficient to result in decreased viral replication and increased immune protection. Correspondingly in humans, uninfected HLA-A*0201+ individuals have an estimated natural SL9 epitope-specific, naïve CTL precursor frequency of approximately one in 3.3×10^6^ cells in the peripheral blood, which is similar to the precursor frequency of naïve cells specific to a variety of other viral antigens [51]. In our studies, the TCR-transduced population typically accounted for 0.75–5.5% of the CD8+ T cell population in a given organ in the mouse following their differentiation from HSCs (the illustration in Figure 1D represents a single mouse from a single experiment). The frequency of transgenic cell reconstitution did not correlate with transduction efficiencies of the vector into the stem cells, rather it appears to be due to individual engraftment rates of CD34+ cells into each mouse. However, even at low frequencies of transgenic TCR expressing cells, this represents a significant increase in the naïve cell precursor frequency for cells specific to the SL9 Gag epitope, as mice harboring the control non-specific TCR and untransduced mice had undetectable levels or very low levels of natural SL9-specific CTLs as determined by MHC tetramer staining. Utilizing TCR gene transductions to yield increases of HIV-specific precursor frequency to conserved antigenic epitopes could potentially reconstitute innate defects in the ability of peripheral T cells to clear infected cells. While the human thymus involutes over time, thus producing fewer T cells in adults than in children, it does retain some activity throughout life [52], [53]. A recent study involving introduction of an antiviral gene into the autologous HSC of HIV infected adults illustrated that naïve T cells bearing the transgene were detected in the peripheral blood of these subjects, indicating that genetically engineered T cells can develop from HSC in adult HIV infected subjects [54]. Through this type of therapeutic intervention, our results suggest the feasibility of supplying newly developed, naïve antigen-reactive cells, that could allow the overall T cell response to overcome limits in the magnitude of the response that inhibit effective viral clearance.

This type of gene therapy-based approach could further diversify the breadth of the responses by naïve, antigen specific cells by utilizing TCRs specific to other epitopes of HIV. The use TCR gene transduction as a therapeutic approach would have to be tailored to the HLA type of the individual receiving treatment in order to produce cells that survive T cell selection processes. Immune epitope escape from the transduced TCR, which did not occur in the time frame of our experiments, is likely to occur *in vivo* in a clinical setting. One potential caveat of the humanized mouse model is the lower level of human immune cell reconstitution than is seen in humans; which significantly, yet incompletely, recapitulated the human immune system in the mouse. While HIV replication rates and viral loads persist detectably over weeks, they do not achieve the levels observed in natural infection in humans. This lower level of viral replication is one potential reason that viral escape mutants to the SL9-specific TCR may be slower to develop. The potential for viral immune escape necessitates the use of multiple TCRs in a therapeutic setting targeted to the antigen or antigens of interest. Careful selection of multiple TCRs targeted to relatively conserved antigenic epitopes within defined HLA types could reduce the possibility of viral epitope evolution and immune escape, perhaps driving the evolution of the virus into a less fit state [55]. The evidence that immune escape and viral evolution against many specific epitopes occurs relatively slowly suggests that an engineered immune response and the immune pressure created by these antigen-specific cells may be therapeutically beneficial by lowing viral replication, decreasing levels of infected cells, and impairing the fitness state of the virus [55], [56]. In sum, our results demonstrate the feasibility of a therapeutic approach that involves the modification of human HSCs by delivering genes for antigen-specific TCR to produce peripheral, naïve, antigen-specific T cells that are capable of reducing HIV replication *in vivo*. These studies provide a foundation and a model system that would allow the closer examination of human antiviral T cell responses and the development of therapeutic strategies that target chronic viral infection.

## Materials and Methods

### Ethics statement

Peripheral blood mononulear cells was obtained at the University of California, Los Angeles in accordance with UCLA Institutional Review Board (IRB) approved protocols under written informed consent using an IRB-approved written consent form by the UCLA Center for AIDS Research Virology Laboratory and Dr. Yang and was distributed for this study without personal identifying information. Human fetal tissue was purchased from Advanced Biosciences Resources or from StemExpress and was obtained without identifying information and did not require IRB approval for its use. Animal research described in this manuscript was performed under the written approval of the UCLA Animal Research Committee (ARC) in accordance to all federal, state, and local guidelines. Specifically, these studies were carried out under strict accordance to the guidelines in The Guide for the Care and Use of Laboratory Animals of the National Institutes of Health and the accreditation and guidelines of the Association for the Assessment and Accreditation of Laboratory Animal Care (AALAC) International under UCLA ARC Protocol Number 2010-038-02B. All surgeries were performed under ketamine/xylazine and isofluorane anesthesia and all efforts were made to minimize animal pain and discomfort.

### Antibodies, tetramers, and flow cytometry

The following antibodies were used in flow cytometry: CD3, CD4, CD11c, CD8, CD45, CD45RA, CD34, HLA-DR (Coulter), CD19, CD56, CCR7, HSA, and IgG controls (eBioscience), hemagglutinin (HA) sequence YPYDVPDYA (Roche), and HLA-A*02 (Serotech). HLA-A*0201 tetramer containing the HIV Gag SL9 SLYNTVATL (SL9) peptide was purchased from Coulter. Cell surface marker expression was analyzed utilizing antibodies conjugated to either fluorescein isothiocyanate (FITC), Peridinin Chlorophyll Protein (PerCP)-Cy5.5, phycoerythrin (PE), electron coupled dye (ECD), PE-Cy5, PE-Cy7, allophycocyanin (APC), APC-Alexa750, APC-eFluor780, Alexa700, eFluor405, Pacific Blue, or Pacific Orange in appropriate combinations. Cells were acquired on a LSR II flow cytometer (BD Biosciences) using FACSDiva software. FlowJo software was used for analysis.

### Lentiviral vector production

Lentiviral production from the plasmid containing the HIV SL9 specific TCR (pCCL.PPT.hPGK.1.9.IRES.eGFP) or a control TCR with an unknown specificity (pCCL.PPT.hPGK.α4.IRES.eGFP) was produced using the Invitrogen ViraPower Lentiviral Expression System using the pCMV.ΔR8.2.Δvpr packaging plasmid and the pCMV-VSV-G envelope protein plasmid as previously described [11].

### Construction of NSG-CTL mice

NSG mice were initially purchased from Jackson Laboratories and bred at the UCLA Division of Laboratory Animal Medicine. To construct NSG-CTL mice, fresh human HLA-A*0201+ fetal liver and thymus pairs from the same donor were obtained from Advanced Biosciences Resources or from StemExpress. Fetal liver was then homogenized and CD34+ cells were isolated as described [11]. Briefly, fetal liver is diced into small (∼3 mm) pieces, homogenized and digested with collagenase type IV (1 mg/ml), hyaluronidase (1 mg/ml), DNase I (2 U/ml)(Sigma). CD34+ cell were purified using magnetic activated cell sorting (Miltenyi). The negative fraction of cells, which contains fetal liver stromal cells (CD34− cells) is saved. CD34+ cells were then genetically transduced following resuspension in Yssel's medium containing 2% human serum albumin and placed in a tissue culture plate coated with 20 µg/ml retronectin (Takara Bio, Inc.) along with lentiviral vector at a multiplicity of infection of 5 overnight at 37°. Fetal liver stromal cells and the matched fetal thymus, cut into small pieces (2 mm), were cultured at 37° overnight in RPMI-1640 containing 10% fetal calf serum (FCS) and 0.44 mg/ml Piptazo. The next day, tissue and cells were washed with PBS and a fraction of the transduced CD34+ cells were then viably frozen. The remaining CD34+ cells were combined with fetal liver stromal cells in cold Matrigel (BD Biosciences) in a 1∶9 ratio (CD34+ cells:fetal liver stromal cells, typically 500,000 transduced CD34+ cells:4,500,00 fetal liver stromal cells) and combined with a 2 mm fetal thymus piece in a trocar and placed under the kidney capsule of NSG mice. Transduction efficiency was determined following culturing in IMDM containing 20%FCS, 50 ng/ml of IL-3, IL-6, and SCF for 3 days, and subsequent assessment of GFP fluorescence by flow cytometry. Transduction efficiency of CD34+ cells occurred at a mean rate of 12.7% (Standard deviation = 12.6%, range 1.63%–38.5%, n = 14). Three weeks following implantation, mice were irradiated with 3 Gy using a cobalt-60 source to clear a niche for human CD34+ cell engraftment of the bone marrow. The frozen CD34+ cells were then thawed and then injected intraveneously into the mice. Mice were then checked for human cell engraftment 6–10 weeks post-injection. Multiple experiments were performed with a minimum of 3 mice per experimental group to yield statistical significance. Each experiment utilized humanized mice that were made from human tissue from same donor and the donor tissue was unique experiment to experiment.

### HIV growth and infection

A virus variant of HIV-1_NL4-3-HSA-HA_ containing the mouse heat stable antigen (HSA) which has been modified to contain the influenza virus hemaggluttin YPYDVPDYA sequence (HA) cloned into the vpr open reading frame, and which also contains the SL9 Gag epitope, has been previously described [18]. Virus was grown in CEMx174 from plasmid-derived virus stock. Viral infectivity was determined by limiting dilution titration on CEMx174 cells. Mice were infected by intraperitoneal injection 10–12 weeks following CD34+ cell injection with 50–100 ng of previously frozen virus stock.

### Reverse-transcriptase, real time PCR for viral RNA in mouse plasma

Mouse blood peripheral blood was drawn by retro-orbital bleeding into glass capillary tubes coated with 330 mM EDTA (Gibco), and 3% sterile human serum albumin (Baxter Healthcare). Viral RNA was extracted from plasma with the High Pure Viral RNA Kit (Roche). The kit is designed to extract 200 µl of plasma. Since there is generally less plasma than this, the volume was estimated by weight and brought up to 200 µl with phosphate buffered saline (Gibco). DNA standards and the template for *in vitro* transcribed RNA for quantitative PCR was derived from pNL101 linearized with EcoRI, checked with electrophoresis, and quantitated by spectrophotometry (A_260_). A section of the gag gene in pNL101 was amplified with the primers NG1CF, position 366–398 (5′-GGAGAATTAGATAAATGGGAAAAAATTCGGTTA-3′) and NG1CR position 679–648 (5′-GCCTTTTTCTTACTTTTGTTTTGCTCTTCCTC-3′), and cloned into pCR4TOPO. The product containing the cloned gag was then digested with SpeI and BsrGI and gel purified. This fragment was then translated to RNA with T7 RNA polymerase (Promega Riboprobe Transcription Kit) and quantitated by spectrophotometry (A_260_). This RNA was serially diluted in The RNA Storage Buffer (Ambion) with 0.4 U/µl Rnasin and 5 ng/µl Lambda DNA/HindIII (as carrier), to make a stock of 500,000 copies/µl. Before each RT run, a fresh vial of RNA was serially diluted in the RNA Storage Buffer (Ambion) to make standards of 100,000 to 10 copies. Quantitative reverse transcriptase-PCR (RT-PCR) was performed using the following primers/probe specific for gag sequences: NG1F (position 453–480) 28 bp (5′-GAGCTAGAACGATTCGCAGTTAATCCTG-3′), NG1R (position 570–534) 37 bp (5′-ATAATGATCTAAGTTCTTCTGATCCTGTCTGAAGGGA-3), NG1Z probe (position 482–520) 39 bp (FAM-5′ -CCTTTTAGAGACATCAGAAGGCTGTAGACAAATACTGGG-3-BHQ).

The final reaction concentration consists of 2.5 µM NG1F, 7.5 µM NG1R, and 2.5 µM NG1Z. These primers were based on sequences identified to be in relatively “open” regions of HIV RNA not impeded by secondary structure interference as determined by [19].

Reverse transcription was performed using the SuperScript III kit (Invitrogen). The annealing step consisted of 5 µl of template RNA plus 3 µl of a mixture consisting of 1.5 parts 20 µM NG1R, 0.5 parts Rnasin plus (Promega), 16 parts 5× RT buffer, and 12 parts water. The resulting 8 µl was heated to 70° for 2 minutes, then at 60° for 5 minutes, then cooled to room temperature. The RT step was run by adding 2 µl of a mixture of 8 parts water, 4 parts 5× buffer, 5 parts DTT, 2 parts 25 mM dNTPs (Invitrogen), and 1 part SuperScript III. This was heated to 55° for 30 minutes, 85° for 5 minutes, then cooled to room temperature.

For quantitative DNA PCR following the reverse transcription step, 15 µl of the PCR mix consisting of 38.5 parts water, 44 parts 25 mM MgCl2, 50 parts NG-FRZ oligos, 5 parts 500 mM Tris buffer pH 8.3, 8.5 parts 1 M KCl, 2.5 parts 25 mM dNTPs, and 1.25 parts Platinum Taq was added to all wells that underwent the reverse transcription reaction and mixed. Real-time, quantitative PCR was performed with 5 minutes activation at 95°, and followed by 45 cycles of 95° for 15 seconds and 60° for one minute on a BioRad CFX96 thermocycler. An additional set of DNA standards, serially diluted from 2×10^5^ copies to 20 copies, of linearized pNL101 was run in parallel to control for the efficiency of the RT step. Results from samples were interpolated within the quantitation derived from the RNA standards.

### Statistical analysis

Statistical support was provided through the UCLA Center for AIDS Research (CFAR) Biostatistical Core. Experiments were analyzed utilizing the Student's t test, the Spearman rank correlation test (SRCT), or the Wilcoxon Rank Sum Test (WRST)(when n = 3), as indicated.

### Sequencing the majority quasispecies from mouse plasma

Evolution/mutation of the dominant version of the introduced Gag-SL9 epitope sequences from the plasma of mice infected with HIV-1_NL4-3-HSA-HA_ was determined by bulk sequencing of the segment of the Gag coding region. Plasma viral RNA was isolated as described above and cDNA was synthesized utilizing the Superscript cDNA synthesis kit (Applied Biosystems). Alternatively, proviral DNA from lymphocytes on infected mice was isolated as described above. Utilizing these DNAs, the region of the HIV-1 Gag flanking the SL9 epitope (a.a. 77–85) was PCR amplified using the 737-Forward primer (5′-GCGACTGGTGAGTACGCC-3′) and the 1255-Reverse primer (5′-ACCCATGCATTTAAAGTTC-3′) and purified by Gel-purification (Qiagen Inc., USA). This purified bulk PCR product was then directly used for dye-terminator sequencing with both 737-Forward and 1255-Reverese primers in parallel. The data was then analyzed by the ABI-3130 genetic analyzer (Applied Biosystems, USA).

## References

[1] Koup RA, Safrit JT, Cao Y, Andrews CA, McLeod G (1994). Temporal association of cellular immune responses with the initial control of viremia in primary human immunodeficiency virus type 1 syndrome.. J Virol.

[2] Ogg GS, Jin X, Bonhoeffer S, Dunbar PR, Nowak MA (1998). Quantitation of HIV-1-specific cytotoxic T lymphocytes and plasma load of viral RNA.. Science.

[3] Borrow P, Lewicki H, Hahn BH, Shaw GM, Oldstone MB (1994). Virus-specific CD8+ cytotoxic T-lymphocyte activity associated with control of viremia in primary human immunodeficiency virus type 1 infection.. J Virol.

[4] Ahlers JD, Belyakov IM (2010). Lessons learned from natural infection: focusing on the design of protective T cell vaccines for HIV/AIDS.. Trends Immunol.

[5] Goonetilleke N, Liu MK, Salazar-Gonzalez JF, Ferrari G, Giorgi E (2009). The first T cell response to transmitted/founder virus contributes to the control of acute viremia in HIV-1 infection.. J Exp Med.

[6] Streeck H, Jolin JS, Qi Y, Yassine-Diab B, Johnson RC (2009). Human immunodeficiency virus type 1-specific CD8+ T-cell responses during primary infection are major determinants of the viral set point and loss of CD4+ T cells.. J Virol.

[7] Li Q, Skinner PJ, Ha SJ, Duan L, Mattila TL (2009). Visualizing antigen-specific and infected cells in situ predicts outcomes in early viral infection.. Science.

[8] Goulder PJ, Watkins DI (2008). Impact of MHC class I diversity on immune control of immunodeficiency virus replication.. Nat Rev Immunol.

[9] Ogg GS, Jin X, Bonhoeffer S, Moss P, Nowak MA (1999). Decay kinetics of human immunodeficiency virus-specific effector cytotoxic T lymphocytes after combination antiretroviral therapy.. J Virol.

[10] Casazza JP, Betts MR, Picker LJ, Koup RA (2001). Decay kinetics of human immunodeficiency virus-specific CD8+ T cells in peripheral blood after initiation of highly active antiretroviral therapy.. J Virol.

[11] Kitchen SG, Bennett M, Galic Z, Kim J, Xu Q (2009). Engineering antigen-specific T cells from genetically modified human hematopoietic stem cells in immunodeficient mice.. PLoS ONE.

[12] Brainard DM, Seung E, Frahm N, Cariappa A, Bailey CC (2009). Induction of Robust Cellular and Humoral Virus-Specific Adaptive Immune Responses in Human Immunodeficiency Virus-Infected Humanized BLT Mice.. J Virol.

[13] Denton PW, Krisko JF, Powell DA, Mathias M, Kwak YT (2010). Systemic administration of antiretrovirals prior to exposure prevents rectal and intravenous HIV-1 transmission in humanized BLT mice.. PLoS ONE.

[14] Denton PW, Estes JD, Sun Z, Othieno FA, Wei BL (2008). Antiretroviral pre-exposure prophylaxis prevents vaginal transmission of HIV-1 in humanized BLT mice.. PLoS Med.

[15] Wege AK, Melkus MW, Denton PW, Estes JD, Garcia JV (2008). Functional and phenotypic characterization of the humanized BLT mouse model.. Curr Top Microbiol Immunol.

[16] Shimizu S, Hong P, Arumugam B, Pokomo L, Boyer J (2010). A highly efficient short hairpin RNA potently down-regulates CCR5 expression in systemic lymphoid organs in the hu-BLT mouse model.. Blood.

[17] Blom B, Spits H (2006). Development of human lymphoid cells.. Annual Rev Immunol.

[18] Ali A, Yang OO (2006). A novel small reporter gene and HIV-1 fitness assay.. J Virol Methods.

[19] Watts JM, Dang KK, Gorelick RJ, Leonard CW, Bess JW (2009). Architecture and secondary structure of an entire HIV-1 RNA genome.. Nature.

[20] Rufer N, Zippelius A, Batard P, Pittet MJ, Kurth I (2003). Ex vivo characterization of human CD8+ T subsets with distinct replicative history and partial effector functions.. Blood.

[21] Romero P, Zippelius A, Kurth I, Pittet MJ, Touvrey C (2007). Four functionally distinct populations of human effector-memory CD8+ T lymphocytes.. J Immunol.

[22] Vatakis DN, Koya RC, Nixon CC, Wei L, Kim SG (2011). Antitumor activity from antigen-specific CD8 T cells generated in vivo from genetically engineered human hematopoietic stem cells.. Proc Natl Acad Sci U S A.

[23] Seder RA, Darrah PA, Roederer M (2008). T-cell quality in memory and protection: implications for vaccine design.. Nat Rev Immunol.

[24] Virgin HW, Wherry EJ, Ahmed R (2009). Redefining chronic viral infection.. Cell.

[25] Jones RB, Ndhlovu LC, Barbour JD, Sheth PM, Jha AR (2008). Tim-3 expression defines a novel population of dysfunctional T cells with highly elevated frequencies in progressive HIV-1 infection.. J Exp Med.

[26] Khaitan A, Unutmaz D (2011). Revisiting immune exhaustion during HIV infection.. Cur HIV/AIDS Rep.

[27] Asquith B, Edwards CT, Lipsitch M, McLean AR (2006). Inefficient cytotoxic T lymphocyte-mediated killing of HIV-1-infected cells in vivo.. PLoS Biol.

[28] Appay V, Nixon DF, Donahoe SM, Gillespie GM, Dong T (2000). HIV-specific CD8(+) T cells produce antiviral cytokines but are impaired in cytolytic function.. J Exp Med.

[29] Kostense S, Ogg GS, Manting EH, Gillespie G, Joling J (2001). High viral burden in the presence of major HIV-specific CD8(+) T cell expansions: evidence for impaired CTL effector function.. Eur J Immunol.

[30] Kadolsky UD, Asquith B (2010). Quantifying the impact of human immunodeficiency virus-1 escape from cytotoxic T-lymphocytes.. PLoS Comput Biol.

[31] Kitchen SG, Jones NR, LaForge S, Whitmire JK, Vu BA (2004). CD4 on CD8+ T cells directly enhances effector function and is a target for HIV infection.. Proc Natl Acad Sci U S A.

[32] Zloza A, Schenkel JM, Tenorio AR, Martinson JA, Jeziorczak PM (2009). Potent HIV-specific responses are enriched in a unique subset of CD8+ T cells that coexpresses CD4 on its surface.. Blood.

[33] Brenchley JM, Hill BJ, Ambrozak DR, Price DA, Guenaga FJ (2004). T-cell subsets that harbor human immunodeficiency virus (HIV) in vivo: implications for HIV pathogenesis.. J Virol.

[34] McCune JM (1992). The SCID-hu mouse: a small animal model for the analysis of human hematolymphoid differentiation and function.. Bone Marrow Transplant.

[35] Stoddart CA, Maidji E, Galkina SA, Kosikova G, Rivera JM (2011). Superior human leukocyte reconstitution and susceptibility to vaginal HIV transmission in humanized NOD-scid IL-2R[gamma]−/− (NSG) BLT mice.. Virology.

[36] Berges BK, Rowan MR (2011). The utility of the new generation of humanized mice to study HIV-1 infection: transmission, prevention, pathogenesis, and treatment.. Retrovirology.

[37] Gorantla S, Makarov E, Finke-Dwyer J, Gebhart CL, Domm W (2010). CD8+ cell depletion accelerates HIV-1 immunopathology in humanized mice.. J Immunol.

[38] Johnson LA, Heemskerk B, Powell DJ, Cohen CJ, Morgan RA (2006). Gene transfer of tumor-reactive TCR confers both high avidity and tumor reactivity to nonreactive peripheral blood mononuclear cells and tumor-infiltrating lymphocytes.. J Immunol.

[39] Hughes MS, Yu YY, Dudley ME, Zheng Z, Robbins PF (2005). Transfer of a TCR gene derived from a patient with a marked antitumor response conveys highly active T-cell effector functions.. Hum Gene Ther.

[40] Clay TM, Custer MC, Sachs J, Hwu P, Rosenberg SA (1999). Efficient transfer of a tumor antigen-reactive TCR to human peripheral blood lymphocytes confers anti-tumor reactivity.. J Immunol.

[41] Joseph A, Zheng JH, Follenzi A, Dilorenzo T, Sango K (2008). Lentiviral vectors encoding human immunodeficiency virus type 1 (HIV-1)-specific T-cell receptor genes efficiently convert peripheral blood CD8 T lymphocytes into cytotoxic T lymphocytes with potent in vitro and in vivo HIV-1-specific inhibitory activity.. J Virol.

[42] Miles JJ, Silins SL, Burrows SR (2006). Engineered T cell receptors and their potential in molecular medicine.. Curr Med Chem.

[43] Cooper LJ, Kalos M, Lewinsohn DA, Riddell SR, Greenberg PD (2000). Transfer of specificity for human immunodeficiency virus type 1 into primary human T lymphocytes by introduction of T-cell receptor genes.. J Virol.

[44] Morgan RA, Dudley ME, Yu YY, Zheng Z, Robbins PF (2003). High efficiency TCR gene transfer into primary human lymphocytes affords avid recognition of melanoma tumor antigen glycoprotein 100 and does not alter the recognition of autologous melanoma antigens.. J Immunol.

[45] Kalos M, Levine BL, Porter DL, Katz S, Grupp SA (2011). T cells with chimeric antigen receptors have potent antitumor effects and can establish memory in patients with advanced leukemia.. Sci Transl Med.

[46] Porter DL, Levine BL, Kalos M, Bagg A, June CH (2011). Chimeric Antigen Receptor-Modified T Cells in Chronic Lymphoid Leukemia.. New Engl J Med.

[47] Morgan RA, Dudley ME, Wunderlich JR, Hughes MS, Yang JC (2006). Cancer regression in patients after transfer of genetically engineered lymphocytes.. Science.

[48] Vatakis DN, Koya RC, Bristol G, Kim S, Liu W (2011). Anti-tumor activity from antigen specific CD8 T cells generated from genetically engineered human hematopoietic stem cells.. Proc Natl Acad Sci U S A.

[49] Kitchen SG, Shimizu S, An DS (2011). Stem cell-based anti-HIV gene therapy.. Virology.

[50] Holt N, Wang J, Kim K, Friedman G, Wang X (2010). Human hematopoietic stem/progenitor cells modified by zinc-finger nucleases targeted to CCR5 control HIV-1 in vivo.. Nat Biotechnol.

[51] Alanio C, Lemaitre F, Law HK, Hasan M, Albert ML (2010). Enumeration of human antigen-specific naive CD8+ T cells reveals conserved precursor frequencies.. Blood.

[52] Douek DC, McFarland RD, Keiser PH, Gage EA, Massey JM (1999). Changes in thymic output with age and during the treatment of HIV infection.. Nature.

[53] Jamieson BD, Douek DC, Killian S, Hultin LE, Scripture-Adams DD (1999). Generation of functional thymocytes in the human adult.. Immunity.

[54] Mitsuyasu R, Merigan TC, Carr A, Zack JA, Winters MA (2009). Phase 2 gene therapy trial of an anti-HIV ribozyme in autologous CD34+ cells.. Nat Med.

[55] Troyer RM, McNevin J, Liu Y, Zhang SC, Krizan RW (2009). Variable fitness impact of HIV-1 escape mutations to cytotoxic T lymphocyte (CTL) response.. PLoS Pathog.

[56] Althaus CL, De Boer RJ (2008). Dynamics of Immune Escape during HIV/SIV Infection.. PLoS Comput Biol.

